# Metabolomic Analysis in Severe Childhood Pneumonia in The Gambia, West Africa: Findings from a Pilot Study

**DOI:** 10.1371/journal.pone.0012655

**Published:** 2010-09-09

**Authors:** Evagelia C. Laiakis, Gerard A. J. Morris, Albert J. Fornace, Stephen R. C. Howie

**Affiliations:** 1 Department of Biochemistry and Molecular and Cellular Biology, and Department of Oncology, Lombardi Comprehensive Cancer Center, Georgetown University, Washington D.C., United States of America; 2 Bacterial Diseases Programme, Medical Research Council (UK) Laboratories, Banjul, The Gambia; 3 WCU Research Center of Nanobiomedical Science, Dankook University, Cheonan, Korea; University of Minnesota, United States of America

## Abstract

**Background:**

Pneumonia remains the leading cause of death in young children globally and improved diagnostics are needed to better identify cases and reduce case fatality. Metabolomics, a rapidly evolving field aimed at characterizing metabolites in biofluids, has the potential to improve diagnostics in a range of diseases. The objective of this pilot study is to apply metabolomic analysis to childhood pneumonia to explore its potential to improve pneumonia diagnosis in a high-burden setting.

**Methodology/Principal Findings:**

Eleven children with World Health Organization (WHO)-defined severe pneumonia of non-homogeneous aetiology were selected in The Gambia, West Africa, along with community controls. Metabolomic analysis of matched plasma and urine samples was undertaken using Ultra Performance Liquid Chromatography (UPLC) coupled to Time-of-Flight Mass Spectrometry (TOFMS). Biomarker extraction was done using SIMCA-P^+^ and Random Forests (RF). ‘Unsupervised’ (blinded) data were analyzed by Principal Component Analysis (PCA), while ‘supervised’ (unblinded) analysis was by Partial Least Squares-Discriminant Analysis (PLS-DA) and Orthogonal Projection to Latent Structures (OPLS). Potential markers were extracted from S-plots constructed following analysis with OPLS, and markers were chosen based on their contribution to the variation and correlation within the data set. The dataset was additionally analyzed with the machine-learning algorithm RF in order to address issues of model overfitting and markers were selected based on their variable importance ranking. Unsupervised PCA analysis revealed good separation of pneumonia and control groups, with even clearer separation of the groups with PLS-DA and OPLS analysis. Statistically significant differences (p<0.05) between groups were seen with the following metabolites: uric acid, hypoxanthine and glutamic acid were higher in plasma from cases, while L-tryptophan and adenosine-5′-diphosphate (ADP) were lower; uric acid and L-histidine were lower in urine from cases. The key limitation of this study is its small size.

**Conclusions/Significance:**

Metabolomic analysis clearly distinguished severe pneumonia patients from community controls. The metabolites identified are important for the host response to infection through antioxidant, inflammatory and antimicrobial pathways, and energy metabolism. Larger studies are needed to determine whether these findings are pneumonia-specific and to distinguish organism-specific responses. Metabolomics has considerable potential to improve diagnostics for childhood pneumonia.

## Introduction

Pneumonia is the biggest single cause of death in children, accounting for around 20% of 10 million deaths under the age of 5 years every year globally, 70% of these occurring in sub-Saharan Africa [1]–[8]. In The Gambia acute lower respiratory infection (ALRI), principally pneumonia, has been documented as the leading cause of death in young children [9], [10]. The global burden of death from pneumonia will need to be markedly reduced if there is to be any prospect of achieving the United Nations' Millennium Development Goal 4 (MDG-4), that is, the reduction of under-5 mortality two-thirds by the year 2015 [11], [12]. International momentum is building to meet this challenge [13].

Case management will remain a key strategy in reducing the mortality of pneumonia, and other infectious diseases, even if current vaccines fulfill their promise. Better diagnostics will be needed to improve case management, the more so as the introduction of conjugate vaccines worldwide changes the aetiology and epidemiology of pneumonia [14], [15]. New laboratory approaches have the potential to deliver improvements in diagnostics and metabolomic analysis is one of these.

Metabolomics is a rapidly evolving field that aims to identify and quantify the concentration changes of all the metabolites (i.e., the metabolome) in a biofluid (e.g. blood, saliva, urine) or model system. This approach has been used successfully to identify biomarkers following exposure to ionizing radiation [16]–[19], metastatic prostate cancer [20]–[23] and assess differences in gut microbiota [24]–[27]. Additionally, it has been utilized to identify biomarkers through Nuclear Magnetic Resonance (NMR) in primarily adult onset pneumonia with known causative agents [28]–[30] and in further elucidation of metabolic pathways of lung injury in mice [31], [32]. Metabolomics has the potential to both improve the understanding of disease mechanisms and the diagnostics. It can be applied to easily accessible biofluids and may offer the eventual possibility of effective non-invasive bedside testing.

This paper describes the application of metabolomic methods in a pilot study to characterize children with and without severe pneumonia. The objective is to obtain preliminary data to assess whether metabolomic analysis might be able to distinguish these groups and hence have potential diagnostic application. It is also hoped that this data might provide pointers for the future exploration of disease mechanisms in childhood pneumonia.

## Methods

### Study Setting, Design, Patient Selection, Consent and Ethical Approval

**Figure 1:**
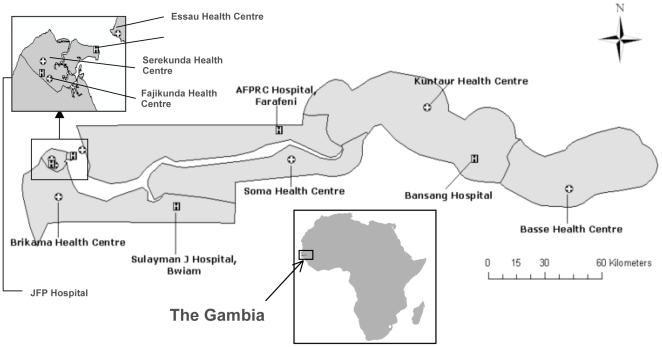
Map of The Gambia, showing hospitals and major health centres and the coastal region in which the study was conducted.

The Gambia is a geographically long and narrow sub-Saharan African country, extending 400 km inland from the West African coast along the Gambia River. It has a population of 1.4 million, over 40% of which is less than 15 years of age (2003 census) [33]. A study of the aetiology of childhood pneumonia is being undertaken in the coastal area of The Gambia (Fig. 1), in which cases of pneumonia are being enrolled along with community controls. The study area comprises Banjul, Kanifing, and Kombo (North, South, Central and East) municipalities. Written informed consent from the parent or guardian is required for inclusion in the study. Specific written informed consent is obtained for percutaneous lung aspiration where the procedure is indicated. The study was approved by the Gambia Government-Medical Research Council Joint Ethics Committee (L2008.28).

### Entry Criteria and Case Definitions

Cases are children aged between 2 and 59 months, originating from within the study area, presenting to the Medical Research Council (MRC) hospital in Fajara, the Royal Victoria Teaching Hospital in Banjul (RVTH), Fajikunda Health Centre, Serekunda Health Centre, or Brikama Health Centre with severe or very severe pneumonia defined clinically by modified World Health Organization (WHO) criteria. Severe pneumonia is defined as cough or difficulty breathing, plus any of lower chest wall indrawing, nasal flaring, or an oxygen saturation of <90% on pulse oximetry (the latter defining very severe pneumonia). Children with a cough of two or more weeks, those with severe anemia (Hb <6 g/dL) and those with confirmed wheeze at recruitment are excluded. Community controls without WHO-defined pneumonia, matched by neighborhood age and sex, are recruited for cases in a 1∶1 ratio.

### Sample Collection, Selection, and Microbiology

Blood from cases was collected for culture and a full blood count, and urine was collected where practicable. Percutaneous lung aspiration for culture was performed where defined safety criteria were met and written informed consent was given. Blood and lung aspirate samples were also subjected to molecular analysis for pathogen detection using *Streptococcus pneumoniae* and *Haemophilus influenzae* specific primers (*lytA* and *cpsA* for *S. pneumoniae*, *glpQ* for *H. influenzae*) and *16SrRNA* primers with sequencing of the gel electrophoresis bands for identification of other bacterial species. The culture and molecular methods used have been described elsewhere [*34*]–[*36*]. On 2 September 2008 one hundred fifteen severe cases had been enrolled in the study of which complete verified data were available for ninety three. Of these, eleven severe pneumonia cases and matched community controls were available that had both plasma and urine samples of sufficient volume for analysis and these were selected for this pilot study. The samples were stored at −40°C and aliquots were shipped on dry ice to Georgetown University for metabolomic analysis.

### Sample Analysis

Urine and plasma samples were analyzed using Ultra Performance Liquid Chromatography (UPLC) coupled to Time-of-Flight Mass Spectrometry (TOFMS) from Waters (Milford, MA). In particular, urine samples were deproteinized by 1∶5 dilution in 50% acetonitrile with 2 mM debrisoquine sulfate and 30 mM 4-nitrobenzoic acid as internal controls. Plasma samples were deproteinized by 1∶40 dilution in 66% acetonitrile containing 2 mM debrisoquine sulfate and 30 mM 4-nitrobenzoic acid as internal controls. Following centrifugation at 13,000× g, 5 mL of the supernatant were injected into the UPLC-TOFMS. A Waters Acquity UPLC BEH C18 2.1×50 mm column packed with 1.7 mm beads was used to separate the molecules in the biofluids set at 40°C for the urine and 60°C for plasma. The mobile phase flow rate was set at 0.5 mL/min. The gradient mobile phase consisted of water with 0.1% formic acid (A) and acetonitrile containing 0.1% formic acid (B). A 10 min urine sample run consisted of 0.5 min of 99% (A), 3.5 min of 20% (B), 4 min of 95% (B), 1 min of 99% (B), and finally 1 min of 99% (A). A 10 min plasma sample run consisted of 0.5 min of 100% of (A), 3.5 min of 60% of (B), 5 min of 100% of (B), and finally 1 min of 100% of (A). Mass spectrometry and accurate mass acquisition was performed with a Waters QTOF Premier® (Milford, MA) operating at either positive-ion (ESI+) or negative-ion (ESI-) electrospray ionization mode. The capillary voltage was set to 3200 V and the sampling cone voltage to 45 V. The desolvation gas flow was set to 800 L/h and the temperature was set to 350°C. The cone gas flow was set to 25 L/h for plasma and 15 L/h for urine and the temperature was set to 130°C. Intermittent injections of sulfadimethoxine as a lock mass ([M+H]^+^ = 311.0814 *m/z* and [M−H]^−^ = 309.0658*m/z*) at a concentration of 300 pg/mL in 50% acetonitrile at a rate of 40 mL/min, were used for accurate mass measurements.

### Data Processing and Multivariate Data Analysis

Mass chromatograms and spectra were acquired with the software MassLynx (Waters) in centroid format and markers were extracted with the software MarkerLynx (Waters). Urine samples were normalized to their respective creatinine relative peak area of [M+H]^+^ = 114.0667 *m/z* with retention time of 0.32 min before further analysis of the data. Two separate multivariate statistical methods were utilized for biomarker extraction, SIMCA-P^+^ vs 12.0 (Umetrics, Sweden) and the machine-learning algorithm Random Forests (RF). For SIMCA-P^+^ analysis, all centroid data were Pareto scaled, which increases the importance of low abundance ions without giving importance to noise. ‘Unsupervised’ (i.e. blinded, identity of the samples was not known by the Georgetown group) data were analyzed by Principal Component Analysis (PCA), while ‘supervised’ (i.e. unblinded, identity of the samples was later revealed) analysis was by Partial Least Squares-Discriminant Analysis (PLS-DA) and Orthogonal Projection to Latent Structures (OPLS). Potential markers were extracted from S-plots constructed following analysis with OPLS, and markers were chosen based on their contribution to the variation and correlation within the data set.

In order to address issues concerning overfitting of the data, which is common with large datasets containing relatively small numbers of samples, Random Forests analysis was performed through R, a programming language that allows for statistical processing [37]. Random Forests is a machine-learning algorithm that has been used successfully in identifying metabolic biomarkers in biofluids [16], [19], [38]. The samples were assigned to control versus pneumonia groups and ten thousand trees were constructed with variable importances averaged over twenty five independent random forests. Multidimensional scaling plots were constructed with analysis of either the whole sample set or the top one hundred metabolites and percentages of classification accuracy were calculated. Bootstrapping of the results from the twenty five independent random trees was applied to determine the 95% confidence intervals of the variable importance ranks. Additionally, heatmaps were designed of the top fifty ions generated through RF. The samples were grouped by treatment and the metabolites were hierarchically clustered by complete linkage using the euclidian distance. To aid in visualization, each metabolite was scaled by the maximum intensity value of that metabolite in the data set (i.e. each row was divided by the maximum value in the row before color assignment).

### Molecular Ion Identification

Selected ions that showed variable differences between the Control and Pneumonia groups were chosen for further analysis and identification. Fifteen ions from either urine or plasma samples were chosen based on the S-plot and RF significance ranking and exhibited statistically significant p-values (p<0.05) based on analysis of the means of the data through two-tailed t-test. Searches for the identity of the metabolites were conducted through the publicly available online database “Madison Metabolomics Consortium Database” (MMCD) with tol (ppm) equal to twenty and ions were validated through tandem mass spectrometry (MS/MS) against pure chemicals (Sigma Aldrich, St. Louis, MO). Chemicals of the highest available purity were either diluted in 50% acetonitrile for urine samples or 66% acetonitrile for plasma samples and fragmented with ramping collision energy of 5–30 eV. MS/MS spectra of the pure chemicals were compared to the biological sample MS/MS spectra for the masses in question.

## Results

Among the eleven children with clinically defined severe pneumonia (the cases), five were female and six were male, and six of the eleven were under two years old while five were two years or older. Ten had radiographic changes of pneumonia, while the other had a normal radiograph. The length of illness at presentation ranged from 1–7 days (median 3 days) and four children had reportedly received antibiotics before presentation. The total white blood count of cases ranged from 7.8 to 67.7×10^3^/mm^3^ (median 13.4) compared to 3.9–11.0 (median 7.2) in community controls. Culture of blood (n = 11) or lung aspirate (n = 3) identified a pathogen in just one case (*S. pneumoniae*) and blood cultures were negative in all controls. Molecular analyses of plasma and lung aspirate identified an organism in seven out of eleven cases: *S. pneumoniae* in five, *H. influenzae* in one and *S. pneumoniae* and *H. influenzae* in the other. Five of the eleven asymptomatic community controls also had organisms identified (*S. pneumoniae* in one, *H. influenzae* in three, *S. pneumoniae* and *H. influenzae* in one).

### Multivariate Data Analysis of Urine Samples

**Figure 2:**
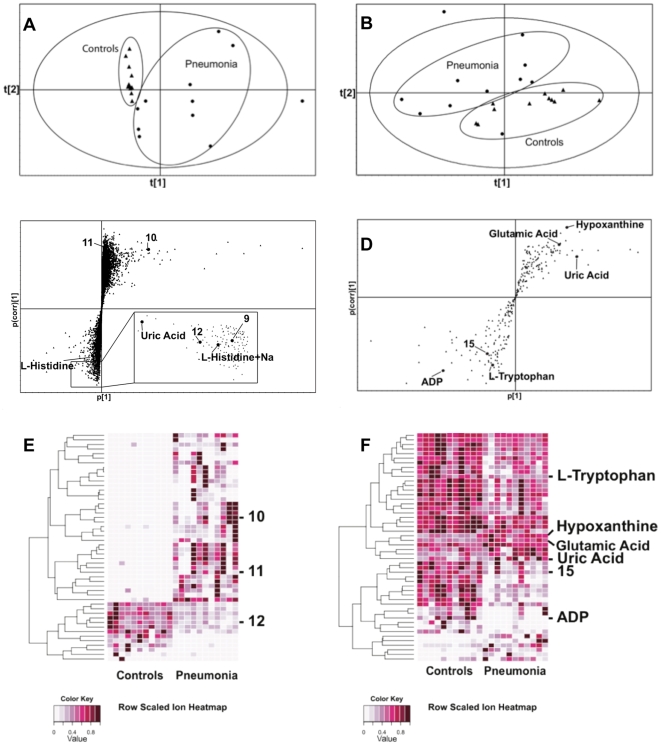
Analysis of the control and pneumonia samples utilizing SIMCA-P^+^ and Random Forests revealed differences in ion abundance between the two groups. Panels A and B show PCA scores plots for urine (ESI+ mode) and for plasma (ESI - mode), respectively. PCA analysis is an unsupervised method of extracting information, where the classes (i.e. experimental groups) are unknown. Panels C and D show the S-plots constructed from the supervised OPLS analysis of urine (ESI+ mode) and plasma (ESI- mode) respectively. Ions with the highest abundance and correlation in the pneumonia group with respect to the controls are present on the upper far right hand quadrant, whereas ions with the lowest abundance and correlation in the pneumonia group with respect to the control group are residing in the lower far left hand quadrant. Ions are marked with either their identity or a number corresponding to Table 1. Panels E and F show heatmaps for urine (ESI+ mode) and plasma (ESI- mode) respectively. The heatmaps were constructed based on the top fifty metabolites of importance, which were extracted with Random Forests analysis. Variable differences are revealed between the control and pneumonia groups, with verified and unknown ions marked on the right corresponding to Table 1. The parallel analysis of the samples with SIMCA-P^+^ and Random Forests allows for the ability to verify that ions, which are identified through both ways (i.e. hypoxanthine), are highly significant, as depicted through two completely different algorithms. Additionally, it allows for the increase of the numbers of ions that are potential candidates for biomarkers.

The urine samples were analyzed in both positive and negative ionization modes with the UPLS-TOFMS. Unsupervised PCA analysis revealed a good separation of the two groups when investigating the first two principal components for the results of positive ionization mode (Fig. 2A). However, a clearer separation of the groups for the negative mode data was seen when the samples were analyzed with PLS-DA (Fig. S1). Additionally, OPLS showed good clustering of the control and pneumonia groups, with larger variation in the pneumonia group (Fig. S1). The S-plots that were constructed from the OPLS analysis showed a significant number of ions up-regulated in the pneumonia group. Additionally, the supervised PLS-DA analysis with assignment of the groups based on disease status and sex revealed a sex dependent clustering of the pneumonia group, which was not present in the controls (Fig. S3). Similar analysis based on age or antibiotic usage within seven days of sample collection did not reveal any clear clustering (data not shown).

RF analysis, utilizing the whole sample set of ions, showed distinct clustering of the two groups in positive mode; however, three of the pneumonia samples were misclassified as belonging in the control group. The overall classification accuracy of the sample set was 86.4%. The negative mode data had an overall classification accuracy of 95.5%, with only one sample of the pneumonia group misclassified as belonging in the controls. A heatmap was additionally constructed based on the top fifty ranked metabolites of the positive mode data, revealing patterns of differential levels of urinary metabolites between controls and pneumonia samples (Fig. 2E).

**Figure 3:**
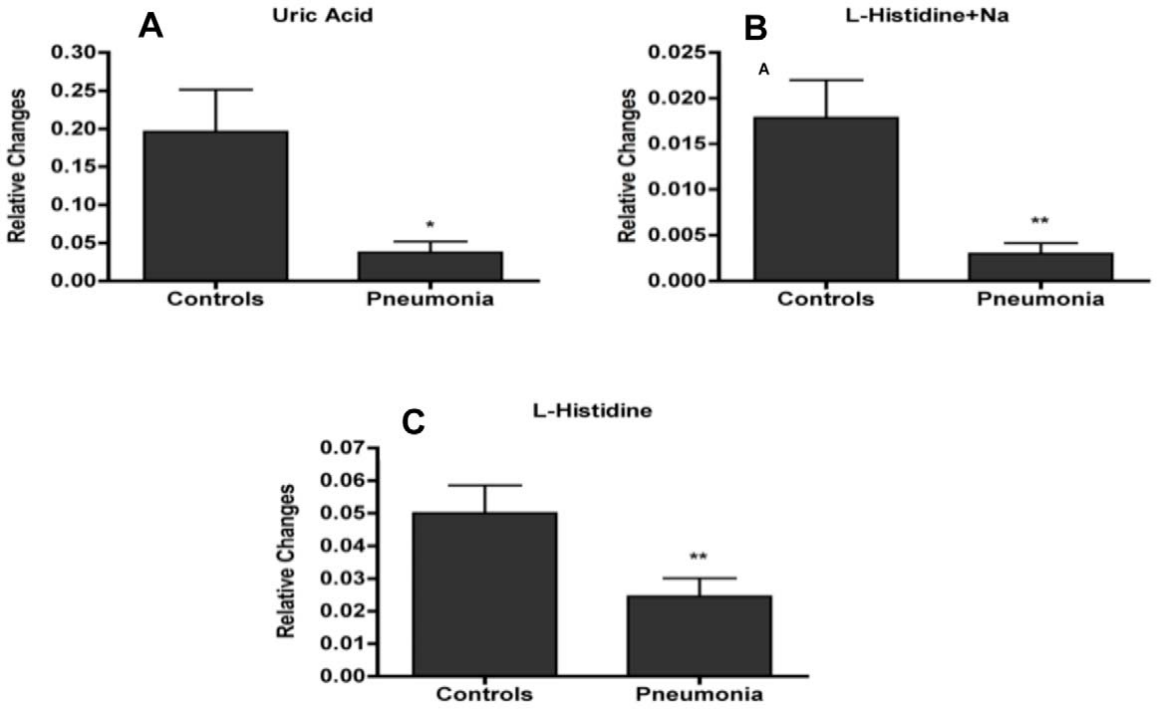
Relative changes of urinary ions that were verified with tandem mass spectrometry. Data is represented at the mean ± SE of the peak areas extracted through the TOFMS data (with * representing p<0.05 and ** representing p<0.01). The real peak areas were normalized to each sample's respective creatinine ([M+H]^+^ = 114.0667 *m/z*) peak area.

Ion selection for verification was based either on the abundance and correlation coordinates of the ions on the S-plot or the importance ranking from RF. Specifically, seven urinary ions were picked based on these criteria for further analysis. Of those, the identity of two ions was verified through MS/MS. Ion 1 from Table 1 with [M+H]^+^ = 169.0352 *m/z* and retention time of 0.3281 min was identified as uric acid, which is involved in the metabolism of purines (Fig. 3A, p = 0.026). Ion 2 with [M+Na]^+^ = 178.0586 *m/z* and retention time of 0.2729 min was verified as L-histidine (Fig. 3B, p = 0.004). Additional search for the protonated form of L-histidine revealed an ion at [M+H]^+^ = 156.078 *m/z* and retention time of 0.2864 min (Fig. 3C, p = 0.00196).

**Table 1:** Identification of urinary and plasma biomarkers in severe pneumonia cases.

				Mass (*m/z*)					
Marker No.	Retention Time (min)	ESI Mode	Biofluid	Found	Calculated	ppm error	Empirical formula	Identity	Relative to Controls
1	0.3281	pos	Urine	169.0352	169.0361	5.3	C5H4N4O3	Uric Acid	Decreased
2	0.2729	pos	Urine	178.0586	178.0592	3.4	C6H9N3O2	L-Histidine+Na	Decreased
2b	0.2864	pos	Urine	156.078	156.0772	5.1	C6H9N3O2	L-Histidine	Decreased
3	0.3179	neg	Plasma	167.0202	167.0205	1.8	C5H4N4O3	Uric Acid	Increased
4	0.3158	neg	Plasma	135.0303	135.0307	3.0	C5H4N4O	Hypoxanthine	Increased
5	0.2821	neg	Plasma	146.0447	146.0453	4.1	C5H8NO4^−^	Glutamic Acid	Increased
6	1.6051	neg	Plasma	203.0811	203.0820	4.4	C11H12N2O2	L-Tryptophan	Decreased
7	0.3533	neg	Plasma	426.0235	426.0216	4.5	C10H15N5O10P2	ADP	Decreased
8	2.764	neg	Urine	-	-	-	-	Unknown	-
9	0.2948	pos	Urine	-	-	-	-	Unknown	-
10	2.3983	pos	Urine	-	-	-	-	Unknown	-
11	3.9999	pos	Urine	-	-	-	-	Unknown	-
12	0.3166	pos	Urine	-	-	-	-	Unknown	-
13	0.317	pos	Plasma	-	-	-	-	Unknown	-
14	0.3231	pos	Plasma	-	-	-	-	Unknown	-
15	0.273	neg	Plasma	-	-	-	-	Unknown	-

Biomarkers that exhibit significant differences between controls and pneumonia samples as determined through SIMCA-P^+^ and Random Forests. Markers 1–7 have been verified through tandem mass spectrometry (MS/MS) against pure chemical standards. Markers 8–15, although were determined as of high importance through the multivariate data analysis, were also tested against pure chemical standards, but were not verified as such.

Five additional urinary ions were further evaluated with MS/MS, though the identities were not verified against the pure chemicals that were tested. In particular ion number 8 ([M−H]^−^ = 145.0606 *m/z*, retention time of 2.764 min), ion number 9 ([M+H]^+^ = 101.0356 *m/z*, retention time of 0.2948 min), ion number 10 ([M+H]^+^ = 335.0676 *m/z*, retention time of 2.3983 min), ion number 11 ([M+H]^+^ = 241.0326 *m/z*, retention time of 3.9999 min), and ion number 12 ([M+H]^+^ = 243.0986 *m/z*, retention time of 0.3166 min) were tested against L-glutamine, alanyl-glycine (Ala-Gly), adipic acid, and D-glutamine for ion 8, hydantoin for ion 9, b-nicotinamide mononucleotide for ion 10, L-cystine for ion 11, and b-thymidine for ion 12. Ions 3–7 and 15 (Table 1) have been clearly marked on Fig. 2D and 2F.

### Multivariate Data Analysis of Plasma Samples

The plasma samples were also analyzed in both positive and negative ionization modes. No normalization was applied to the samples, unlike the urines, since the levels of the plasma that were obtained were tightly controlled. Analysis of the positive ionization mode data with the multivariate statistical software SIMCA-P^+^ demonstrated a good separation between the two groups at the PCA scores plot (Fig. S2). Only one sample of the control group was misclassified as belonging in the pneumonia group, meaning that its overall metabolite profile resembled closely that of the pneumonia group. The separation became clearer when supervised PLS-DA analysis was applied, demonstrating the variable differences between the two groups (Fig. S2). On the negative ionization mode, the PCA analysis also revealed a clear separation between the two experimental groups, with only one pneumonia sample being misclassified as belonging in the control group (Fig. 2B). Furthermore, the PLS-DA and OPLS scores plots revealed a distinct separation of controls from pneumonia samples (Fig. S2). For further analysis of ions, the negative mode S-plot was chosen for extraction of markers (Fig. 2D). RF analysis showed an overall classification accuracy of 86.4%, which increased to 90.9% when only the top one hundred ions were used to classify the samples into separate groups. The percentages of classification accuracy were identical for the analysis of both positive and negative mode data.

**Figure 4:**
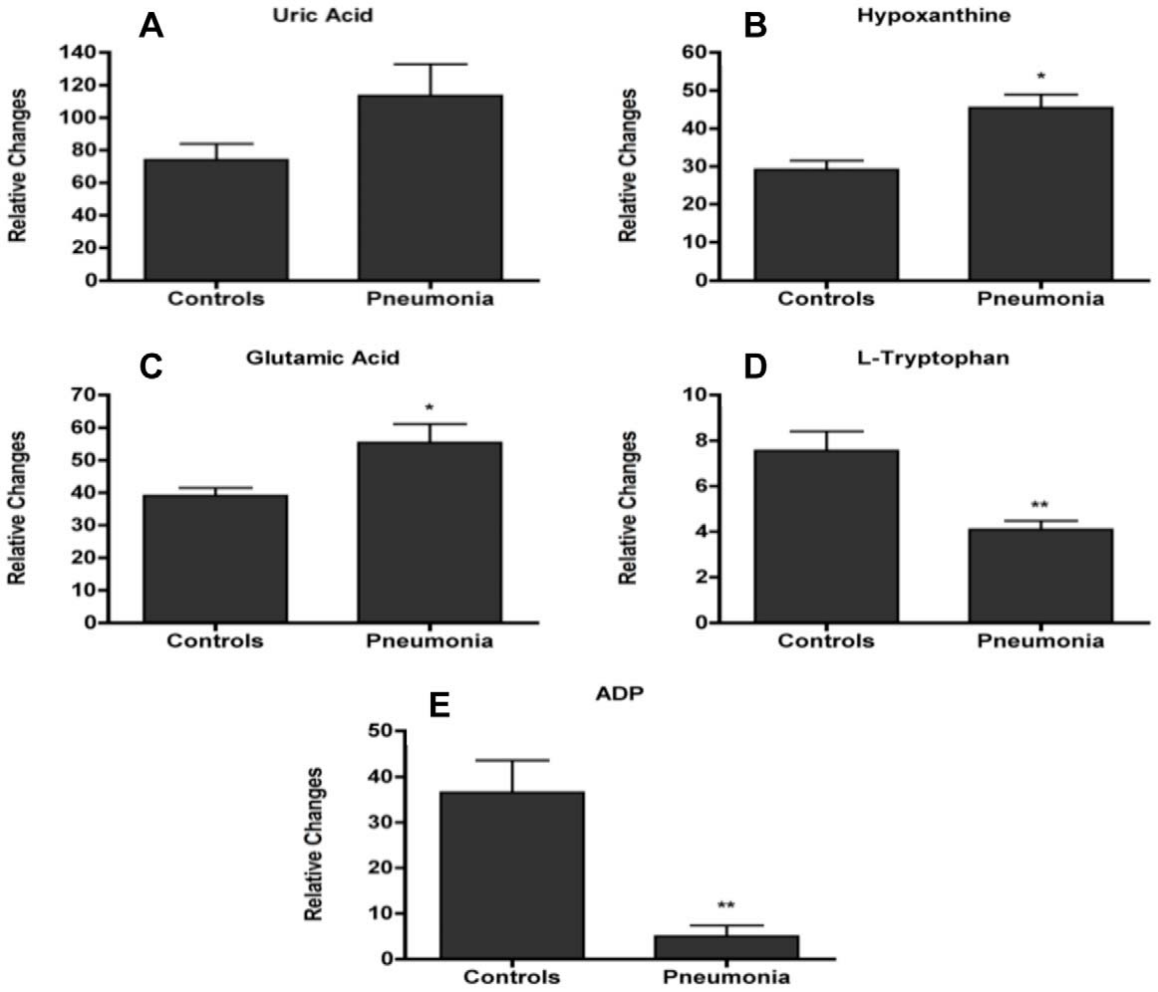
Relative changes of the plasma ions that were verified with tandem mass spectrometry. Data is represented as mean ± SE of the peak areas extracted through the TOFMS data (with * representing p<0.05 and ** representing p<0.01). Unlike the urine samples, plasma samples do not require normalization to a particular metabolite since the volumes obtained are tightly controlled. Uric acid (Panel A) is upregulated in pneumonia plasma levels, although not statistically significant (p = 0.119). This is in contrast to the urine findings. Hypoxanthine and glutamic acid levels in Panels B and C are significantly upregulated in pneumonia samples. On the other hand, L-tryptophan and adenosine-5′-diphosphate (ADP) in Panels D and E are significantly downregulated in pneumonia samples.

Selected ions for further verification and validation were chosen through the S-plots and RF top ranked lists. In particular, ion number 3 with [M−H]^−^ = 167.0202 *m/z*, retention time of 0.3179 min was verified as uric acid, which as mentioned earlier is involved in the purine metabolism. Plasma levels of uric acid appear to be upregulated in the pneumonia group relative to the controls, although not statistically significant (Fig. 4A, p = 0.119). This is in contrast to the urine levels where the uric acid levels are downregulated in the pneumonia group (Fig. 3A, p = 0.026). Ion number 4 with [M−H]^−^ = 135.0303 *m/z*, retention time of 0.3158 min was verified to be hypoxanthine, also involved in the metabolism of purines (Fig. 4B, p = 0.008). Ion number 5 with [M−H]^−^ = 146.0447 *m/z*, retention time of 0.2821 min was verified to be glutamic acid, involved in multiple metabolic processes (Fig. 4C, p = 0.0165). Ion number 6 with [M−H]^−^ = 203.0811 *m/z*, retention time of 1.6051 min was verified to be L-tryptophan, which is an essential amino acid and the only stereoisomer used in structural or enzymatic proteins (Fig. 4D, p = 0.006). Ion number 7 with [M−H]^−^ = 426.0235 *m/z* and retention time of 0.3533 min was verified to be adenosine-5′-diphosphate (ADP) (Fig. 4E, p = 0.0004).

Four additional ions were further investigated with MS/MS against pure chemicals; however, their identity was not verified as the MS/MS spectra differed significantly. In particular ion number 13 ([M+H]^+^ = 274.0924 *m/*z, retention time of 0.317 min), ion number 14 ([M+H]^+^ = 112.0496 *m/*z, retention time of 0.3231 min), and ion number 15 ([M−H]^−^ = 145.0604 *m/z*, retention time of 0.273 min) were tested against 2′-deoxyadenosine and muramic acid for ion 13, cytosine and pyrrole-2-carboxylic acid for ion 14, and L-glutamine, Ala-Gly, adipic acid, and D-glutamine for ion 15. Ions 3–6, 14 and 15 (Table 1) have clearly been marked on both the S-plot and the heatmap (Fig. 4D and F).

## Discussion

Metabolomics is a powerful new technology that allows for the assessment of global metabolic profiles in easily accessible biofluids and biomarker discovery in order to distinguish between diseased and non-diseased status. We utilized this approach in a pilot study in urine and plasma samples from pneumonia patients from The Gambia. The global metabolic profiling and subsequent multivariate analysis clearly distinguished severe pneumonia patients from matched community controls. Although no common pathogenic factor was identified in all the cases, it is noteworthy that a similar disease manifestation allows for similar metabolic profiles and identification of biomarkers. Six metabolites emerged as markers of key differences between the two groups: uric acid, L-histidine, hypoxanthine, glutamic acid, L-tryptophan, and ADP. These metabolites together are important for the host response to infection through antioxidant, inflammatory and antimicrobial pathways, and energy metabolism.

Our observation of lower levels of urinary uric acid in severe pneumonia patients relative to controls suggests increased tubular reabsorption and renal retention of the analyte perhaps to protect against oxidative stress [39], [40]. In contrast, plasma levels of uric acid were elevated in pneumonia cases. In *in vitro* studies, uric acid reacted rapidly with ozone and conferred protection of plasma lipids from peroxidation and erythrocyte lysis [39]. Additional studies have shown that uric acid released from injured cells constitutes a major endogenous danger signal that activates the NALP3 inflammasome (also called cryopyrin or NLRP3), leading to IL-1b production [41] as part of the host response to lung inflammation and fibrosis. Taken together, these studies suggest that uric acid plays a major anti-inflammatory role in pneumonia cases and allows for protection of the host organism from oxidative damage. Additionally, plasma hypoxanthine levels were elevated in patients relative to controls. Hypoxanthine and xanthine are the precursors of uric acid and uric acid was also elevated in the plasma of pneumonia patients, although not statistically significant. Xanthine on the other hand was not identified as a marker through the statistical analysis. High concentrations of hypoxanthine, xanthine, and uric acid have also been shown in patients with bacterial meningitis [42]. This may be because sepsis provokes significant alterations in energy metabolism homeostasis with hypoxanthine and uric acid, offering possibly useful surrogate markers of infection [42], [43]. Elevation of hypoxanthine has also been reported during septic shock and may reflect early high energy nucleotide failure [44].

An additional marker with potentially important implications for disease outcome is ADP. The main role of ADP in the blood is the activation of platelets for effective hemostasis and blood aggregation [45], [46]. Our results indicated that ADP levels in plasma from pneumonia patients are downregulated. This in turn may lead to decreased platelet activation and decreased formation of aggregates and thromboemboli. Lack of purinergic receptors for ADP is a possible way to protect against aggregate formation, however the reduced plasma levels of ADP in pneumonia patients in our studies could confer a protective mechanism against organ failure [45], [47]. Nonetheless, other mechanisms, such as chemokine activation [46], appear to mediate the platelet activation under certain conditions, such as low ADP availability. Further work is required in this direction of low plasma ADP in pneumonia cases and its role in disease outcome and patient survival.

The last three markers identified in this study are amino acids. Hendriksen et al stressed that glutamic acid uptake and synthesis is important for full *Streptococcus pneumoniae* fitness and virulence [48]. The higher levels of glutamic acid in the plasma in patients relative to controls in our study may indicate cellular injury and protection. Additionally, excess circulation of glutamic acid during the disease state requires special attention in this particular human population as it can be associated with central nervous system damage, which is sometimes associated with severe pneumonia [49]. L-tryptophan, on the other hand, was lower in plasma of patients relative to controls. Tryptophan starvation is a recognized antimicrobial defense mechanism through Indoleamine 2,3-dioxygenase (IDO) and mediates immunoregulatory effects [50]. It is possible therefore, that tryptophan starvation initially exhibits an antimicrobial effect to aid in fighting the disease status and later contributes in regulating the T-cell response from a possible overstimulation [50]. Urinary L-histidine was also lower in patients relative to controls. L-histidine is a precursor for the synthesis of histamine, a major contributor to inflammation, asthma, and potentially pneumonia in both human [51] and animal studies [52]. The lack on detection of histamine in urines of pneumonia patients and retention of L-histidine is possible indication that histidine is being converted to histamine in the tissues of pneumonia patients and contributing to their inflammatory state and propagation of disease status.

The sex dependent clustering of the pneumonia group, which was not present in the controls, points to differences in the metabolic response to pneumonia between male and female patients. Sex differences have been documented in survival following community-acquired pneumonia and nosocomial infections, which could be explained by differences in immune responses, genetics, or sex hormone levels [53], [54]. Additionally, a study by Casimir et al on childhood pneumonia revealed significant differences in inflammatory markers between male and female patients [55], making identification of metabolic differences between male and female patients an attractive candidate for future studies on diagnosis and drug development.

This small-scale preliminary study has clear limitations. It is not possible to say whether the metabolomic profile seen in these children with severe pneumonia is pneumonia-specific or associated with a wider spectrum of illness. Either of these possibilities is potentially diagnostically significant, and further work investigating the specificity of the findings must be done to resolve this question. The literature reporting metabolomic analysis in infectious diseases is limited. The majority of the work has been conducted in meningitis patients and considerable work has been conducted on the assessment of global metabolic profiling of bacteria [56]–[58]. Pneumonia specific urinary metabolomic studies have concentrated on primarily adult populations with specific aetiology; however, this study is the first to provide pneumonia metabolomic analysis in urine and plasma from a specific pediatric population in parallel. Additionally, the differences in markers identified could be attributed to age and population related differences, overall aetiology of the pneumonia phenotype, and technologies and analytical methods utilized. This study was too small to define organism-specific metabolic responses, which will be useful for diagnosis, and it was not possible using available sensitive molecular techniques to distinguish causative pathogens from asymptomatic ‘DNAemia’. This is a general challenge for the growing field of molecular diagnostics rather than a limitation of this study in particular. The size of this study also means it has likely failed to identify other metabolites that will be important for diagnostics in the future.

The ability of the methods used in this study to clearly distinguish the children with severe pneumonia from their controls points to the considerable potential of metabolomics to improve diagnosis in sick children and to advance the knowledge of disease mechanisms. This preliminary work's importance is further emphasized by the fact that specific markers were identified in an outbred human population with genetic variability, no clear common causative agent, and simply a shared clinical syndrome. Metabolomics may provide an effective means to overcome the inability of current molecular pathogen detection techniques to distinguish causative pathogens from organisms that are ‘innocent bystanders’. Larger scale studies are now needed to determine the extent of its potential and to identify markers for different causative agents and for other potentially important aspects of disease such as illness severity, key comorbidities, and response to treatment. Once a panel of key biomarkers has been established there is the potential to take metabolomics closer to the bedside through its incorporation into point-of-care devices, which it is hoped will deliver breakthroughs in care in high mortality settings, and the evolution of which will likely be rapid in the next few years.

## Supporting Information

Figure S1 Scores plots generated through the chemometric software SIMCA-P+ vs 12.0. A and B depict the separation between the controls and pneumonia groups when assessing the metabolic profile of urines under positive ionization mode. A is a plot generated from PLS-DA analysis whereas B is an OPLS plot, from which we are able to determine that a greater variability exists within the pneumonia group, possibly due to different aetiological agents. C, D, and E panels in order represent the PCA, PLS-DA and OPLS plots of the negative ionization mode urines.
(0.36 MB TIF)

Figure S2 Scores plots generated through SIMCA-P+ based on the metabolic profiles of plasma samples from controls and pneumonia patients. A, B, and C panels show the PCA, PLS-DA and OPLS scores plots from positive ionization mode plasma samples, respectively. D and E show the PLS-DA and OPLS scores plots from the negative ionization mode.
(0.37 MB TIF)

Figure S3 PLS-DA scores plots examining the existence of sex differences based on metabolic profiles between control and pneumonia groups. Panels A (ESI+) and B (ESI-) based on urinary metabolic profiles clearly depict that possible differences based on sex could exist in the pneumonia group. In panels C (ESI+) of plasma samples separation of profiles based on sex is still evident, whereas in panel D (ESI-) of plasma samples the separation is not existent.
(0.38 MB TIF)
