# Supplementary figures and images for: Metabolomic Analysis in Severe Childhood Pneumonia in The Gambia, West Africa: Findings from a Pilot Study
Source: PLoS One. 2010 Sep 9;5(9):e12655. doi: 10.1371/journal.pone.0012655 (PMC2936566; doi:10.1371/journal.pone.0012655)

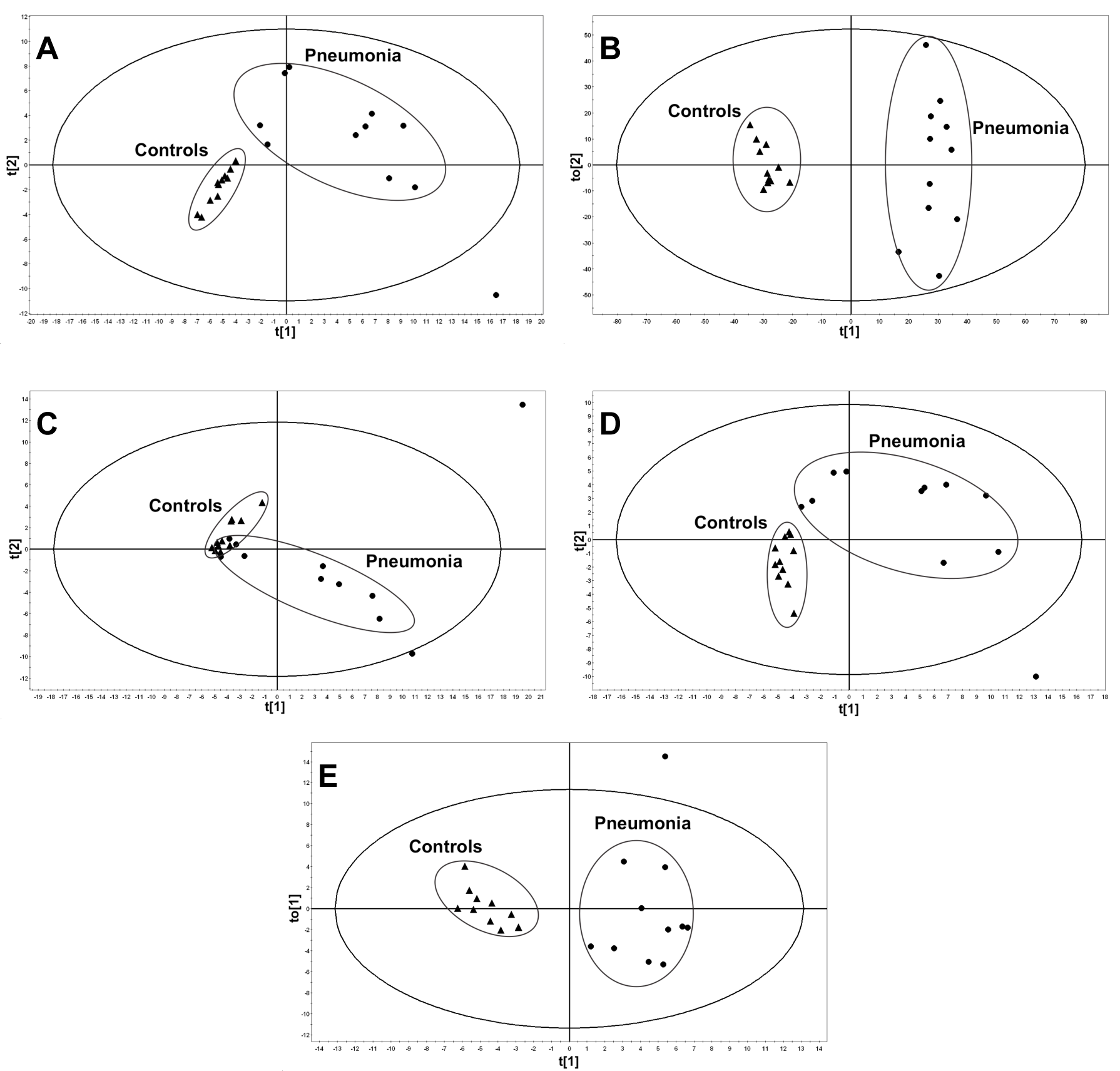

Supplement: Figure S1 — Scores plots generated through the chemometric software SIMCA-P+ vs 12.0. A and B depict the separation between the controls and pneumonia groups when assessing the metabolic profile of urines under positive ionization mode. A is a plot generated from PLS-DA analysis whereas B is an OPLS plot, from which we are able to determine that a greater variability exists within the pneumonia group, possibly due to different aetiological agents. C, D, and E panels in order represent the PCA, PLS-DA and OPLS plots of the negative ionization mode urines. (0.36 MB TIF) [file pone.0012655.s001.tif]

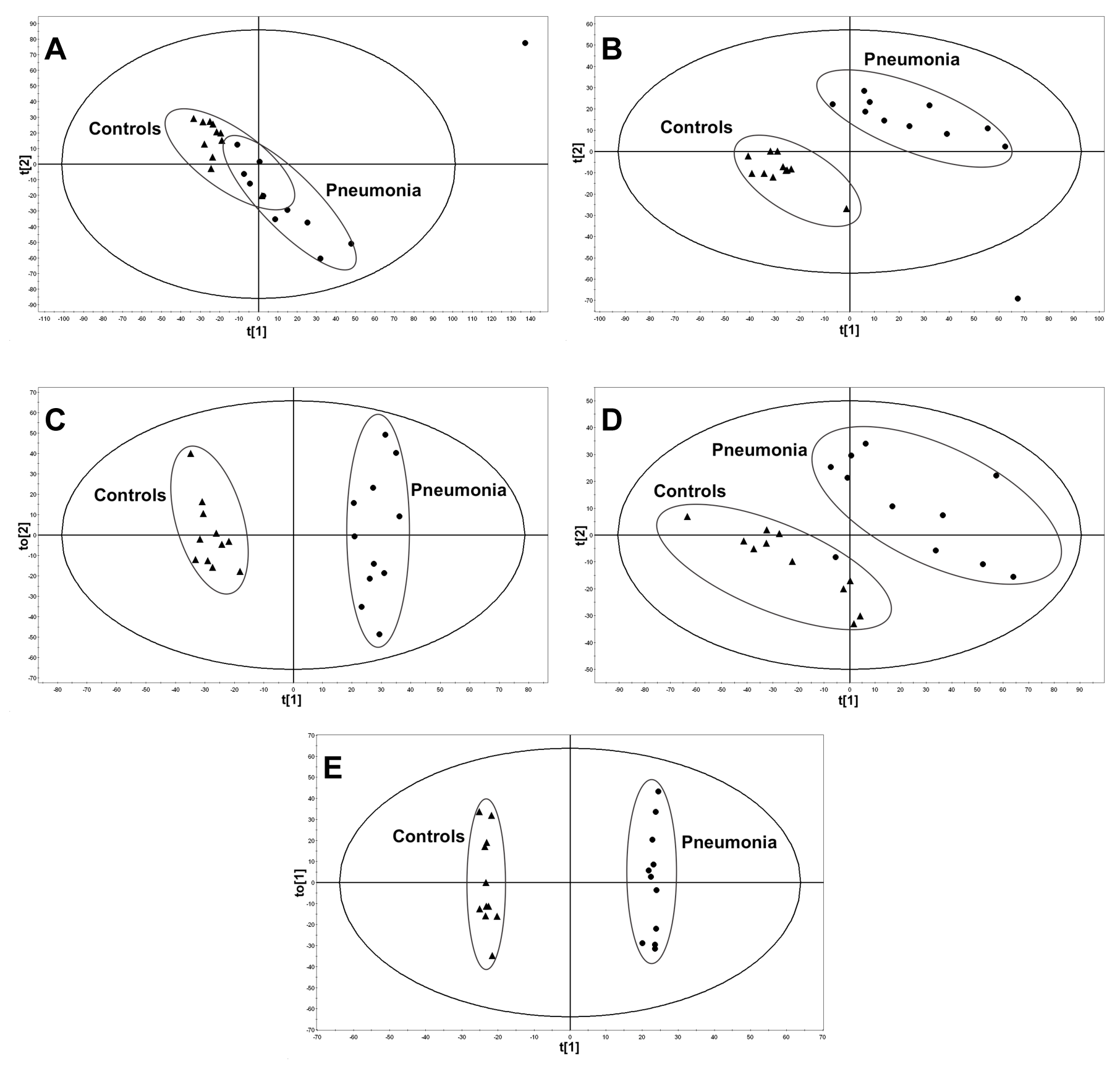

Supplement: Figure S2 — Scores plots generated through SIMCA-P+ based on the metabolic profiles of plasma samples from controls and pneumonia patients. A, B, and C panels show the PCA, PLS-DA and OPLS scores plots from positive ionization mode plasma samples, respectively. D and E show the PLS-DA and OPLS scores plots from the negative ionization mode. (0.37 MB TIF) [file pone.0012655.s002.tif]

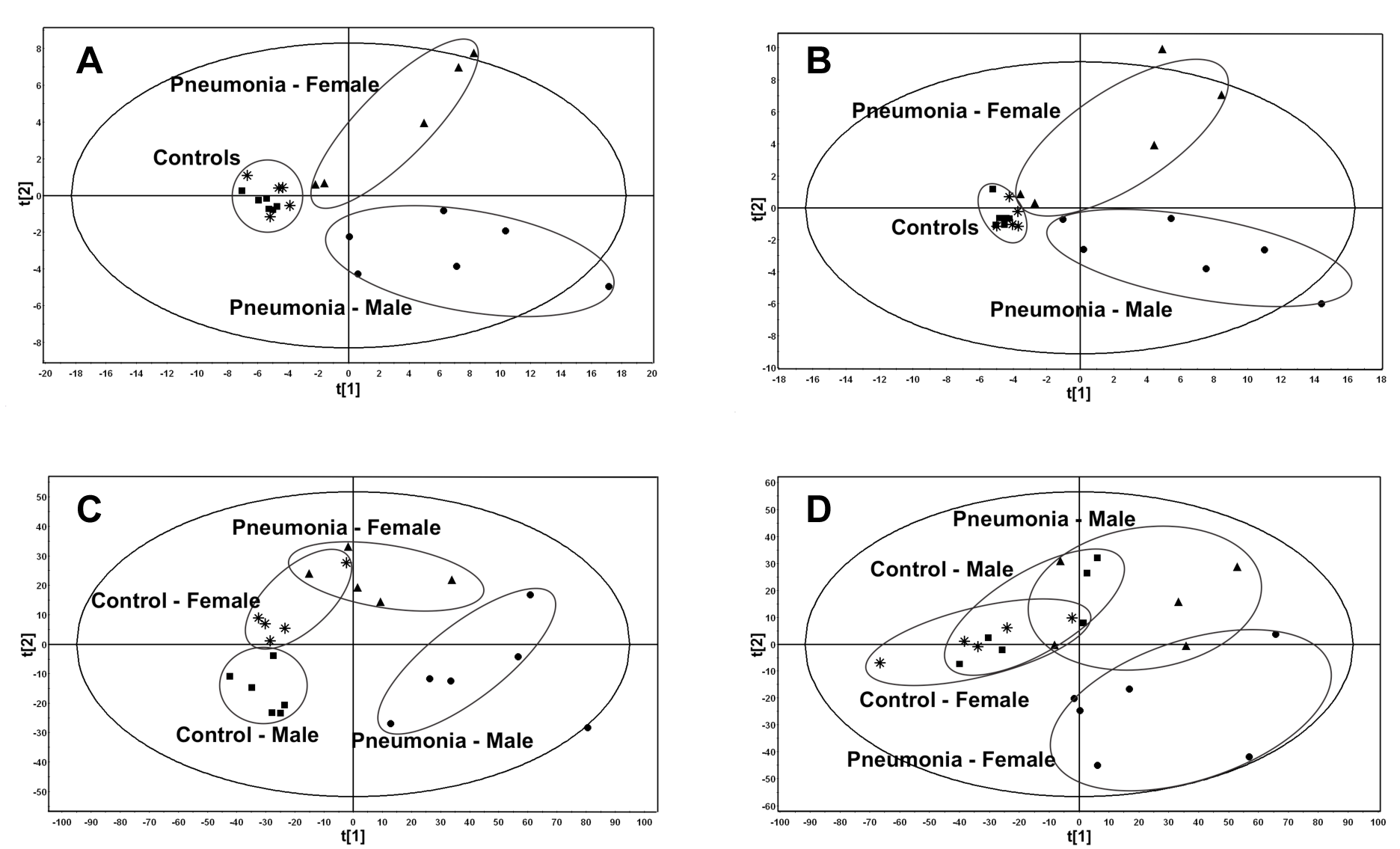

Supplement: Figure S3 — PLS-DA scores plots examining the existence of sex differences based on metabolic profiles between control and pneumonia groups. Panels A (ESI+) and B (ESI-) based on urinary metabolic profiles clearly depict that possible differences based on sex could exist in the pneumonia group. In panels C (ESI+) of plasma samples separation of profiles based on sex is still evident, whereas in panel D (ESI-) of plasma samples the separation is not existent. (0.38 MB TIF) [file pone.0012655.s003.tif]
